# Advanced Iterative Metal Artifact Reduction With Intrinsic Spectral Information From Photon-counting Detector Computed Tomography: Improving the Assessment of Metal-bone Interface

**DOI:** 10.1097/RLI.0000000000001282

**Published:** 2026-02-25

**Authors:** Tristan T. Demmert, Thilo Schikorra, Sandro-Michael Heining, Andreas Specovius, Konstantin Klambauer, Lukas J. Moser, Victor Mergen, Bernhard Schmidt, Thomas Flohr, Matthias Eberhard, Hatem Alkadhi

**Affiliations:** Computed Tomography Division, Siemens Healthineers AG, Forchheim, Germany (T.T.D., T.S., K.K., L.M., V.M., T.F., M.E., H.A.); Department of Radiology, University Hospital Erlangen, Friedrich-Alexander University Erlangen-Nuernberg, Erlangen, Germany (T.S.); Department of Traumatology, University Hospital Zurich, University of Zurich, Zurich, Switzerland (S-M.H.); Siemens Healthineers AG, Forchheim, Germany (A.S., B.S.); Department of Radiology and Nuclear Medicine, Maastricht University Medical Centre+, Maastricht, The Netherlands (T.F.)

**Keywords:** computed tomography, photon-counting detector, metal artifact reduction, fixation nail implant

## Abstract

**Background:**

Metallic implants can cause relevant artifacts in computed tomography (CT) imaging, affecting the quality and diagnostic utility of scans. Previous advancements in metal artifact reduction techniques have shown promise but still exhibit limitations in artifact reduction, particularly close to metal implants.

**Purpose:**

To evaluate a novel, advanced iterative metal artifact reduction (iMAR) algorithm for photon-counting detector CT in an experimental study focused on visualizing the vicinity of a fixation nail implant.

**Methods:**

Three bovine femur bones with titanium-based trochanteric fixation nail implants were scanned on a clinical photon-counting detector CT scanner. Images were reconstructed (1) without iMAR, (2) with the current iMAR algorithm, and (3) with a new prototype iMAR algorithm. The new iMAR prototype algorithm advances state-of-the-art iMAR for photon-counting detector CT by utilizing intrinsically available spectral information. Attenuation and artifact severity (SD of attenuation) were quantified by placing regions-of-interest on each reconstruction across 3 different axial slices: One in the bone marrow immediately adjacent to the metal implant and one in the water adjacent to the femur with the implant. Qualitative image quality, newly introduced artifacts, and diagnostic confidence were rated by 3 radiologists using 5-point Likert scales. Differences between reconstructions were tested using the Friedman test with Wilcoxon post hoc tests; interreader agreement was assessed using Krippendorff alpha.

**Results:**

Artifact severity in the bone adjacent to the implant significantly decreased from 226 HU (no iMAR) to 174 HU (current iMAR) and to 159 HU with the new iMAR (*P* < 0.001). Adjacent to the femur, artifact severity decreased from 63 HU to 48 HU and to 29 HU, respectively (*P* < 0.05). Qualitative scores differed significantly between reconstructions (*P* < 0.05), with highest ratings for new iMAR across all categories. Current iMAR introduced new artifacts near the implant, which did not occur with new iMAR (*P* < 0.05).

**Conclusion:**

Experimental evidence from a bovine femur implant model suggests that a new, advanced iterative metal artifact reduction algorithm leveraging intrinsic spectral information from photon-counting detector CT effectively reduces metal artifacts and further improves the visualization of the metal-bone interface. Thus, this technique has the potential to enhance the assessment of implant-related complications such as aseptic loosening.

Metallic implants lead to artifacts in computed tomography (CT) imaging caused by numerous factors such as scatter, beam hardening, photon starvation, noise, and edge effects.^1^ Another factor influencing the degree of metal artifacts is the atomic number of the respective implant. Metals with high atomic numbers such as stainless steel produce worse artifacts compared with metals with low atomic numbers such as titanium.^2^ Still, even titanium implants may cause considerable artifacts, decrease image quality, and potentially obscure clinical information.

Different approaches have been developed to reduce metal artifacts in CT, beginning in 1987 with Kalender et al^3^ introducing metal artifact reduction (MAR) through linear sinogram interpolation, the basis of metal artifact reduction algorithms. Since then, substantial improvements led to the normalized MAR^4^ and today’s state-of-the-art frequency-split MAR.^5^ Other approaches include replacing artifacts with smooth or interpolated data,^6–8^ applying iterative^9,10^ and deep learning techniques,^11^ and using virtual monoenergetic images at high energy levels.^12^

Despite these technological advances, even the latest attempts to reduce metal artifacts have shortcomings: Spatial resolution may be reduced, and the algorithms themselves can introduce new artifacts that may mimic pathologies such as implant fractures or even malignant lesions.^13–16^ Areas close to the metal are particularly prone to artifacts,^1^ which is critical in the clinical setting, given that aseptic loosening represents the most frequent reason for revision surgery after hip arthroplasty.^17^

The aim of this experimental study was to evaluate a new, advanced iterative metal artifact reduction (iMAR) algorithm for photon-counting detector CT in an experimental study focusing on visualizing the vicinity of metal of a fixation nail implant.

## METHODS

### Phantom

Three bovine femur bones without soft tissue were bought from a local butcher. A senior orthopedic physician implanted trochanteric fixation nails advanced (TFNA, Johnson & Johnson GmbH) with a diameter of 10 mm into the 3 femur bones (Fig. 1). The medullary canal of each femur was initially accessed by opening the cortex with a 16 mm drill. Thereafter, the canal was prepared with different drill diameters depending on the desired implant fit. After the opening, the first nail was driven into the left femur bone diaphysis without a predrilled hole to simulate a perfect fitting implant (bone 1). For the second nail, a hole of 14.5 mm was predrilled into the medullary canal of a right femur to simulate slight implant loosening (bone 2). The hole predrilled into the third left femur had a diameter of 19 mm to simulate more extensive implant loosening (bone 3). Before scanning, the femur bones with the implants were placed in a plastic box (60x30 cm) filled with water to simulate human attenuation (Fig. 1).

**FIGURE 1:**
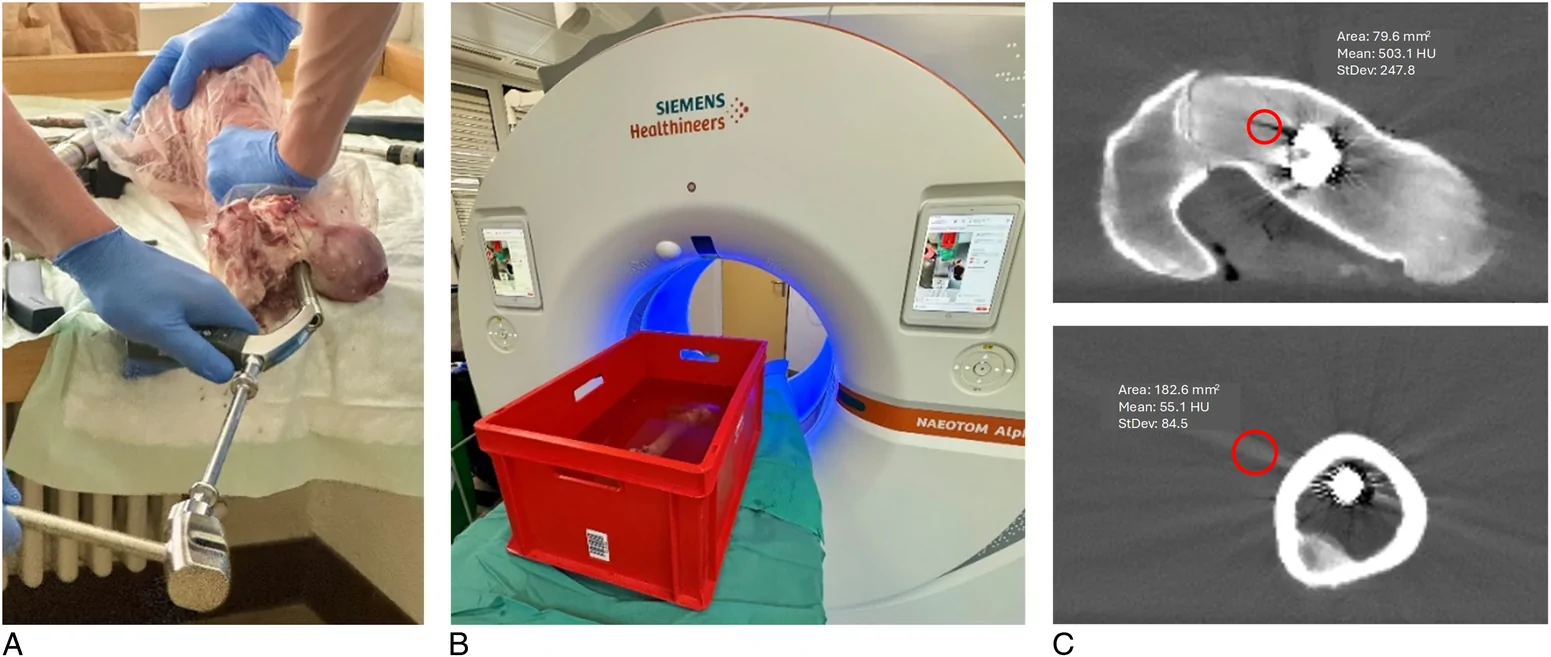
A, Photograph of the implantation procedure of the nail into the bovine femur. B, Positioning of the femur in the water-filled box during scanning. C, Illustration of the region-of-interest placements immediately adjacent to the implant (top) and adjacent to the femur (bottom).

#### Computed Tomography Data Acquisition

Each femur was scanned on a first-generation dual-source photon-counting detector CT machine (NAEOTOM Alpha, version VB10A, Siemens Healthineers AG) with our institutional pelvis protocol and the following parameters: Tube voltage 140 kVp and image quality (IQ) level 55, corresponding to a tube current-time product of 28 mAs and a computed tomography dose index (CTDI) of 3.18 mGy (IQR: 3.0 to 3.3 mGy). The detector collimation was 144×0.4 mm, and the pitch factor was 0.8. Each femur was scanned twice: Aligned parallel to the *z*-axis on the CT scanner table and slightly tilted by 10 to 15 degrees to simulate the range of possible positioning of human lower extremities during scanning.

#### Computed Tomography Data Reconstruction

The scans were reconstructed as virtual monoenergetic images at 55 keV using 3 different approaches:

- At a slice width of 2.0 mm, increment of 1.6 mm, employing a sharp quantitative kernel (Qr64).
- With the same settings and using the state-of-the-art iMAR algorithm of the CT scanner^10,18^ with the hip implant preset.
- With the same settings and using a new, advanced prototype iMAR algorithm, also with the hip implant preset.

All reconstructions were performed using a prototype software (ReconCT v.2025.9.0.0, Siemens).

#### Novel Iterative Metal Artifact Reduction Algorithm for Photon-counting Detector Computed Tomography

The advanced iMAR prototype algorithm investigated in this work extends the state-of-the-art iMAR algorithm with spectral processing and thus represents an advancement of the state-of-the-art iMAR algorithm for photon-counting CT.

The state-of-the-art iMAR algorithm implements a normalized MAR [4] step and thus requires both, spatial information about where metal is contained in the image and a so-called prior image for the normalization step of normalized MAR, which enables preservation of image information away from metals during the inpainting step of normalized MAR. Typically, both are obtained directly from and individually for the actual images through HU-value thresholding. The quality of this information depends on the actual level of image artifacts and can thus also potentially vary for different acquisition spectra.

Photon-counting detector CT provides geometrically fully consistent spectral information, which is utilized in the prototype algorithm to compute generic enhanced prior information in the following way:

The available spectral channels are reconstructed and mixed, which allows to create mixed images that feature suppressed metal artifacts. Operating on these artifact-suppressed mixed images with the same techniques as used with state-of-the-art iMAR enables better metal identification and better prior image derivation. Both data products are then used as shared generic prior information for state-of-the-art iMAR per spectral channel, superseding the individual channel-wise derivation of these data products.

However, direct use of the generic prior image calculated from the mixed image for normalized MAR on an actual spectral channel is prevented by the fact that materials that feature a strong energy dependency in their photon attenuation properties in the for CT relevant energy range significantly differ in their HU-value between the spectral channels and thus also between the spectral channels and the mixed image. Thus, for the proper application of the generic prior image on a spectral channel, it needs to be adjusted for the actual spectral channel acquisition spectrum. By assuming that there are only air, water, and calcium involved, the necessary adjustment can be achieved by rescaling the HU-values within bone-like regions of the prior image with the help of known calcium enhancements per spectral channel and the corresponding enhancement in the mixed image.

Operating on the described combination of the available spectra facilitates the following 2 major improvements with a positive impact on the artifact reduction performance of the iMAR algorithm:

First, metal structures can be identified more reliably. In fact, the correct spatial identification of metal objects in the images is crucial for metal artifact reduction since that spatial information directly impacts sinogram interpolation. In state-of-the-art iMAR, metal objects are identified through thresholding the input images. Metal-induced artifacts such as blooming, hyperdense band, or streaking artifacts complicate the identification of true metal objects. These hyperdense artifacts can happen to be misidentified as metal, resulting in a reduced performance of consecutive artifact reduction steps. Performing metal identification on the mixed, metal artifact-suppressed images allows enhanced metal identification performance.

Second, enhanced material classification of nonmetals can be achieved. The normalization step of the normalized MAR component of the iMAR algorithm requires a material classification of every voxel as air-like, tissue-like, or bone-like. Metal artifacts can lead to misclassificatio,n which results in a degraded performance of the artifact reduction. Performing such material classification on the mixed, metal artifact-suppressed images allows enhanced classification performance.

The prototypic iMAR algorithm investigated here was developed and provided by the vendor and might be made available on photon-counting detector CT scanners in the future.

#### Image Analysis

***Quantitative Analysis***: Circular regions-of-interest (ROI) were placed on each reconstruction from each of the 3 femurs in 3 axial slices based on predefined anatomic levels (Fig. 1):

- At the level of the femoral neck.
- At the level of the proximal femoral shaft.
- At the level of the distal femoral shaft.

At each anatomic level, we placed 2 ROIs:

- Immediately adjacent to the metal implant in the bone marrow in regions with hypoattenuating artifacts (one ROI per slice, average area 80 mm^2^).
- In the water immediately adjacent to the femur with the implant in regions with both hyper- and hypoattenuating artifacts (one ROI per slice, average area 183 mm^2^).

In total, 108 ROIs have been placed in areas with visually pronounced metal-induced artifacts. Attenuation values of the ROIs adjacent to the implant were not used for comparative analysis because they are strongly biased by metal artifacts and their correction; therefore, attenuation was evaluated only in the ROIs placed in water adjacent to the femur. The ROI size, shape, and positioning were kept identical across all reconstructions from the same scan by copying the ROIs between data sets. For each ROI, the average attenuation (in Hounsfield Units, HU) and the SD of the attenuation, the latter as an indicator of artifact severity, were noted. Artifact severity was defined as the SD of attenuation, as previously described in the literature for quantitative assessment of metal artifacts.^19–22^ A reference-based approach using differences to visually artifact-free tissue was not applied, because in our phantom, the bone marrow was globally affected by metal artifacts in the no-iMAR reconstructions, and no reliable artifact-free reference region could be defined.

***Qualitative Analysis:*** Three radiologists (with 2, 5, and 15 y of experience in radiology, respectively) independently evaluated the image data, blinded to the reconstruction method and to each other’s results, with 5-point Likert Scale as previously shown:^21,23^ Overall image quality (1 = nondiagnostic, 2 = poor, 3 = fair/moderate artifacts, 4 = good/minor artifacts, 5 = excellent/no artifacts), severity of newly introduced artifacts through the iMAR algorithms (1 = massive artifacts, 2 = pronounced artifacts, 3 = moderate artifacts, 4 = minor artifacts, 5 = no artifacts), and diagnostic confidence considering the evaluation of potential pathologies close to the metallic implant such as loosening (1 = insufficient, 2 = restricted, 3 = hampered, 4 = marginally affected, 5 = full diagnostic quality).

During evaluation, readers were allowed to adjust window setting at their discretion, starting with a preset window setting of width 2000 HU and center 350 HU.

### Statistical Analysis

The mean and the SD of the attenuation were calculated for each algorithm and femur separately. For statistical testing, measurements from multiple ROIs and slices were averaged per femur and reconstruction, and the femur was used as the statistical unit to avoid pseudo-replication. To evaluate the effect of the orientation of the femur on the reconstructions, we compared the attenuation and artifact severity between the straight and the oblique positioned femurs with the Wilcoxon signed rank test. The differences between the 3 reconstruction methods (no iMAR, current iMAR, new iMAR) in attenuation and artifact severity were tested with the Friedman test. We compared both the artifact severity close to the implant and adjacent to the bone. For all significant results, post hoc analyses were performed using Wilcoxon signed rank tests with the Holm-Bonferroni correction.

Interreader agreement for the ordinal endpoints (overall image quality, severity of new artifacts, interpretability of bone adjacent to the implant) was quantified with Krippendorff alpha (ordinal); 95% CIs were obtained by bootstrap. Alpha values were interpreted according to Landis and Koch^24^: <0 poor, 0.00 to 0.20 slight, 0.21 to 0.40 fair, 0.41 to 0.60 moderate, 0.61 to 0.80 substantial, and 0.81 to 1.00 almost perfect agreement. The effect of femur orientation (straight vs oblique) on qualitative assessment was evaluated with the paired Wilcoxon signed-rank test (2-sided). Scores between reconstructions (no iMAR, current iMAR, new iMAR) were tested with a Friedman test, followed—if significant—by pairwise Wilcoxon tests with Holm adjustment. For the severity of new artifacts, no iMAR had any ratings; therefore, only the paired comparison between the current iMAR versus new iMAR was performed (exact paired Wilcoxon signed-rank test). In addition, an exploratory Spearman rank correlation was performed between quantitative artifact severity and qualitative image quality scores. Statistical significance was set at α = 0.05; all tests were nonparametric and appropriate for ordinal data with possible missing values. All statistical tests, calculations, and graphs were created with commercial software (R Statistical Software, v4.5.1; R Core Team 2025).

## RESULTS

### Quantitative Image Quality Analysis

Quantitative results for each scan and reconstruction method are listed in Table 1. There were no significant differences between the artifact severity in the straight and oblique positioned femur (*P* = 0.587). Similarly, the attenuation in all ROIs was not significantly different between the straight and oblique positioned femur (*P* = 0.322). Consequently, we averaged all quantitative image quality measures for both femur positions for the following analyses.

**TABLE 1:** Attenuation and Artifact Severity Close to the Implant and Adjacent to the Femur for All Scans, Reconstructions, and Bones

		Adjacent to the Implant	Adjacent to the Femur	
Scan	iMAR Algorithm	Artifact Severity (SD in HU)]	Attenuation (HU)	Artifact Severity (SD in HU)
Femur 1 straight	None	281	44	62
	Current	218	16	36
	New	206	9	28
Femur 1 oblique	None	232	9	80
	Current	201	76	53
	New	176	9	31
Femur 2 straight	None	220	16	57
	Current	175	44	56
	New	158	8	27
Femur 2 oblique	None	182	4	58
	Current	150	9	61
	New	138	1	32
Femur 3 straight	None	187	26	65
	Current	129	12	39
	New	116	13	30
Femur 3 oblique	None	255	31	58
	Current	174	39	41
	New	160	13	28

Data are shown as mean values over the 3 ROIs in each reconstruction.

HU indicates Hounsfield Units; iMAR, iterative metal artifact reduction.

The artifact severity, quantified by the SD of the attenuation, close to the metal implant, was significantly different between the 3 reconstruction methods (*P* < 0.001). Post hoc analysis revealed significant differences between no iMAR (mean: 226 HU ± 107) and current iMAR (mean: 174 HU ± 89), between no and new iMAR (mean: 159 HU ± 99), and between current and the new iMAR (all, *P* < 0.001; Fig. 2).

**FIGURE 2:**
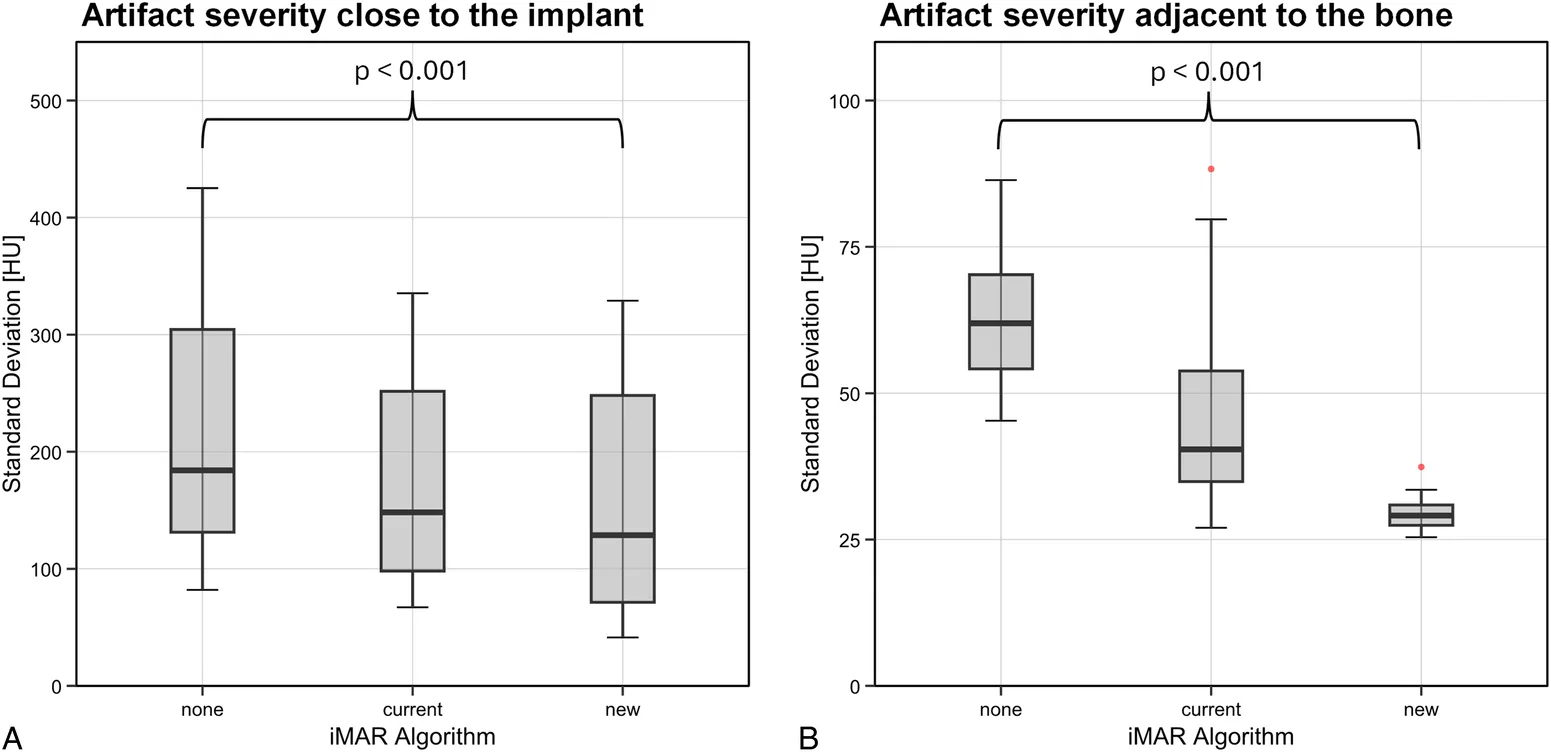
Comparison of the artifact severity in bone immediately adjacent to the implant (A), and adjacent to the bone (B) across all scans, in reconstructions without iterative metal artifact reduction (iMAR), with current iMAR, and with the new iMAR algorithm. The boxes show medians, first quartile, and third quartile borders. Whiskers show the minimum and maximum values within 1.5 times the interquartile range. Outliers are marked as points. *P* values indicate the results from the overall Friedman test comparing all 3 reconstruction methods (no iMAR, current iMAR, and new iMAR).

The analysis of artifact severity adjacent to the femur showed similar results: There were significant differences in artifact severity between the reconstructions (*P* < 0.001). Post hoc comparisons showed that the artifact severity was significantly higher in reconstructions without iMAR (mean: 63 HU ± 12) compared with the current iMAR (mean: 48 HU ± 18) and the new iMAR (mean: 29 HU ± 3), and higher in the current compared with the new iMAR (all, *P* < 0.05; Fig. 2). With new iMAR, the variability of the artifact severity was smaller than in the reconstructions with no and with current iMAR.

The attenuation adjacent to the femur was not significantly different between reconstructions (*P* = 0.056; Fig. 3). Attenuation adjacent to the femur was closest to 0 HU, representing the attenuation of water in the images reconstructed when using the new iMAR algorithm.

**FIGURE 3:**
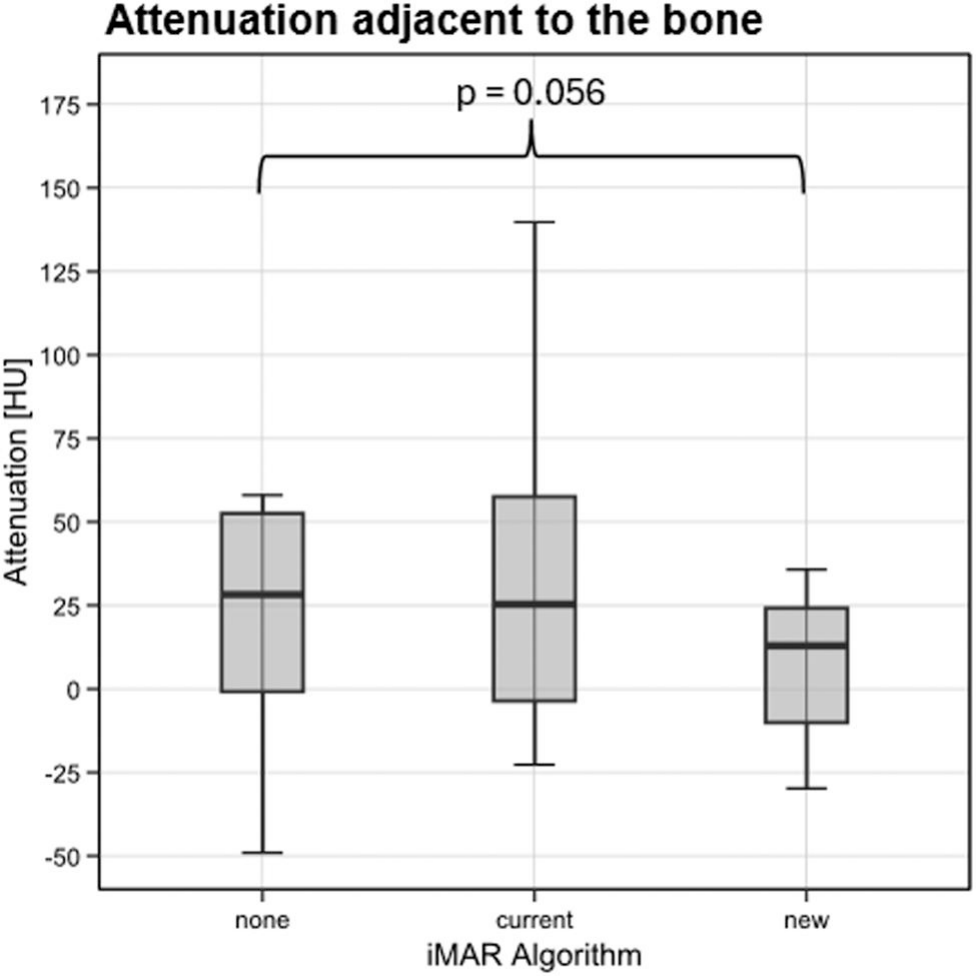
Comparison of the attenuation in adjacent to the femur across all scans, in reconstructions without iMAR, with current iMAR, and with the new iMAR algorithm. The boxes show medians, first quartile, and third quartile borders. Whiskers show the minimum and maximum values within 1.5 times the interquartile range. Outliers are marked as points. *P*-values of the performed Friedman tests are displayed.

### Qualitative Image Quality Analysis

Interreader agreement was good to excellent (Krippendorff α: overall: 0.763, 95% CI: 0.62-0.86; severity of new artifacts: 0.907, 95% CI: 0.85-0.97; interpretability of bone: 0.828, 95% CI: 0.72-0.90; pooled α: 0.848, 95% CI: 0.791-0.885). Femur orientation (straight vs oblique) had no measurable effect on any qualitative endpoint (paired Wilcoxon, all *P* > 0.05).

Across reconstructions, differences were significant for the overall image quality, severity of new artifacts, and interpretability of bone adjacent to the implant (Friedman, *P* < 0.05). Post hoc paired Wilcoxon tests with Holm adjustment consistently showed the highest ratings for new iMAR, being lower for current iMAR, and lowest for no iMAR (Fig. 4). For overall image quality, all 3 pairwise comparisons reached statistical significance after adjustment (*P* < 0.05). For the interpretability of bone adjacent to the metal, new iMAR and current iMAR were both significantly better than no iMAR, with new iMAR outperforming current iMAR. New artifacts were significantly more often induced by current as compared with new iMAR (*P* < 0.05).

**FIGURE 4:**
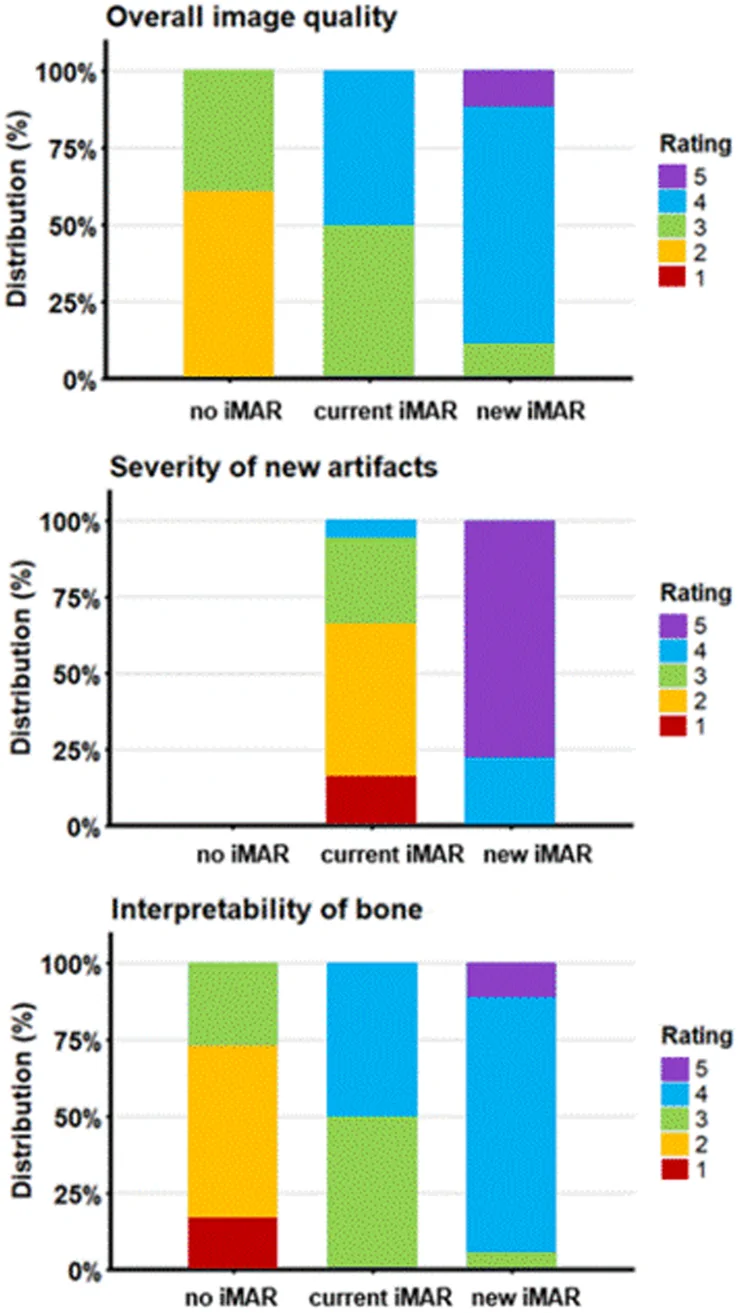
Qualitative image quality ratings across all scans, reconstructions, and femurs. Data are shown as columns representing the distribution of all reader ratings across all 6 scans. Ratings were performed using three 5-point Likert scales as defined in the Methods section: overall image quality (1=nondiagnostic, 5=excellent/no artifacts), severity of newly introduced artifacts (1=massive artifacts, 5=no artifacts), and diagnostic interpretability of the bone adjacent to the implant (1=insufficient, 5=full diagnostic quality).

Spearman correlation analysis showed an inverse association between quantitative artifact severity and qualitative image quality, reaching statistical significance for measurements in water (*P* < 0.05) and showing a similar inverse trend for bone marrow measurements.

Representative examples of the 3 reconstruction methods illustrating typical artifact patterns are shown in Figure 5.

**FIGURE 5:**
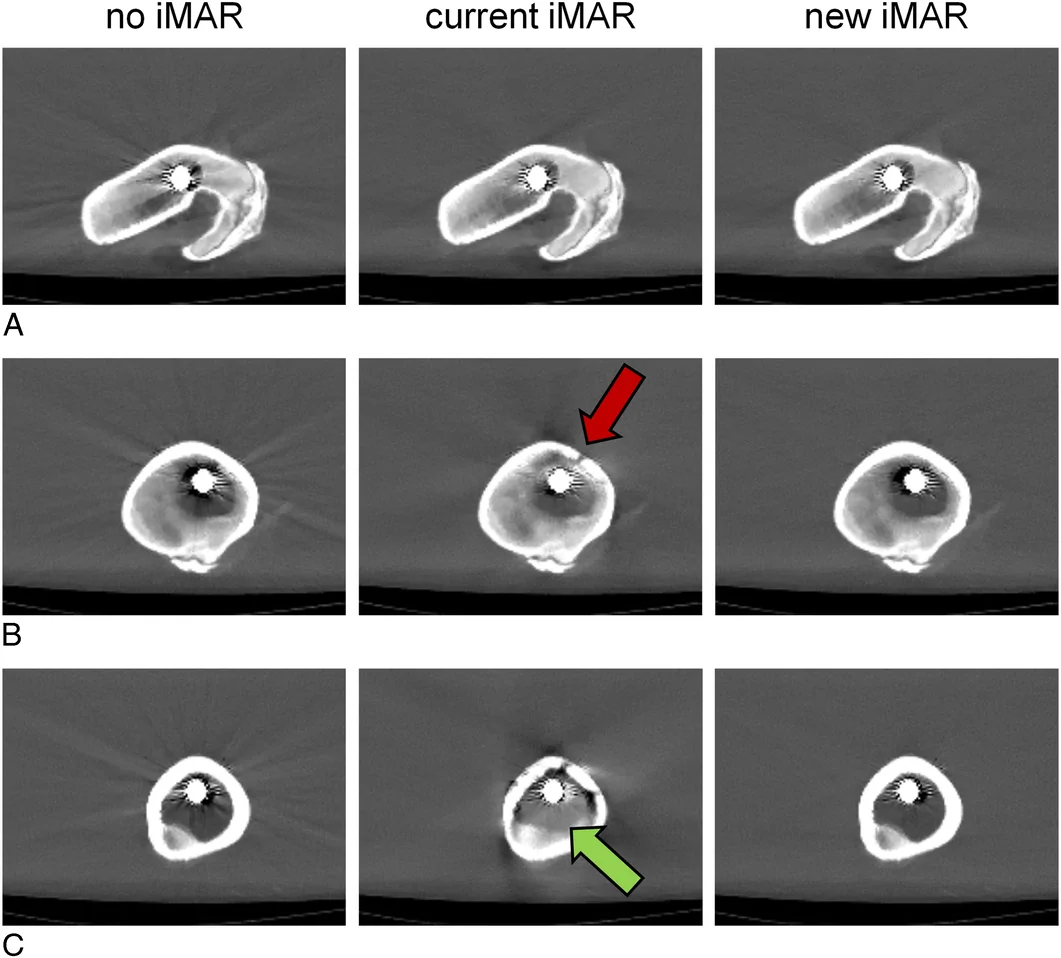
A–C, Representative images of the 3 reconstructions at 3 different axial slices (femoral neck, proximal femoral shaft, and distal femoral shaft) of the same straight-positioned femur with same window settings. Left column shows images without iMAR, middle column with the current iMAR, and right column with the new iMAR. Note the newly introduced artifacts indicating a potential fracture in the cortical bone (red arrow) and the increased attenuation in bone marrow (green arrow) adjacent to the metal in the reconstruction with current iMAR.

## DISCUSSION

This experimental study evaluated a novel prototypic iterative metal artifact reduction (iMAR) algorithm advancement for photon-counting CT. The new iMAR algorithm significantly reduced metal-related artifacts compared with both reconstructions without iMAR and with the currently available clinical iMAR. Improvements were most pronounced in the region immediately adjacent to the metallic implant, where conventional methods typically perform less effectively and, in some cases, even introduce new artifacts. Quantitative analysis confirmed lower artifact burden, while qualitative assessments demonstrated higher overall image quality and diagnostic confidence without the introduction of new artifacts by the new algorithm.

### Comparison With Previous Work on Iterative Metal Artifact Reduction

Previous investigations have shown that iterative reconstruction techniques reduce metal artifacts in conventional CT, but with variable effectiveness depending on implant type and scanning parameters.^25,26^ Morsbach et al^18^ demonstrated that iterative reconstruction improved visualization of fixation nail implants compared with filtered back projection, while Reinert et al^27^ and Pallasch et al^23^ reported enhanced diagnostic quality in clinical settings. Skornitzke et al^10^ further confirmed in a phantom study with metal inserts that the current iMAR algorithm reduces artifacts in photon-counting detector CT. Recent work has further refined iterative metal artifact reduction by introducing modular post-correction strategies applicable to both energy-integrating and photon-counting CT systems.^28^ Nevertheless, residual artifacts remain in areas directly adjacent to metal, where beam hardening and photon starvation effects are most pronounced.^10^

### Limitations of Current Iterative Metal Artifact Reduction Algorithms

Despite their overall effectiveness, current iMAR algorithms show characteristic limitations. Wellenberg et al^13^ reported that even though iterative approaches reduce streaking artifacts in regions distant from the implant, they can generate new artifacts or image distortions close to metal. After iMAR processing, Park et al^15^ described artificial findings mimicking hardware loosening, illustrating that algorithm-induced alterations may affect diagnostic interpretation. These limitations result from interactions in dense material regions and insufficient modeling of complex scatter conditions at the implant interface.

### Implications of the New Iterative Metal Artifact Reduction Approach

The prototypic iMAR algorithm advancement evaluated in this study was developed to beneficially utilize the spectral information available with photon counting CT for metal artifact reduction. The improvements implemented in the prototype have the potential to improve artifact reduction performance, especially in the proximity to metal objects.

Nevertheless, the interface between metal and surrounding structures is still challenging even for the improved iMAR algorithm and should always be assessed with caution. Still, our results demonstrate fewer artifacts near the implant compared with current iMAR, while avoiding newly introduced image distortions, which occurred in images reconstructed with current iMAR. The improved performance at the metal–bone interface has the potential to enable a more reliable assessment of periprosthetic structures, which is clinically relevant for the detection of aseptic loosening and other implant-associated complications.^29^

Beyond this immediate application, the new iMAR algorithm holds potential for use in other clinical areas as well.^30^ In radiotherapy planning, the accurate depiction of metal implants is critical for precise dose calculations. Metal-induced artifacts can distort CT-based attenuation maps, leading to errors in radiation dose delivery.^31^ It was previously shown that iterative MAR algorithms improve dosimetric accuracy by reducing these artifacts, and the enhanced performance of the new iMAR algorithm could further optimize radiotherapy planning in patients with implants.^32^

Metal-induced artifacts also play a major role in head and neck CT imaging, where dental hardware frequently degrades image quality.^33^ Recent evidence from photon-counting CT demonstrates that advanced MAR techniques can substantially mitigate these limitations. Pallasch et al^23^ reported that several metal artifact reduction strategies in first-generation photon-counting CT significantly improved image quality and reduced streak artifacts in patients with dental hardware, thereby enhancing the diagnostic assessment of adjacent tissues. Similarly, Patzer et al^34^ showed that the combination of virtual monoenergetic imaging with iterative MAR techniques further improves artifact suppression around dental implants, allowing for better delineation of critical anatomic structures. These findings indicate that the improved artifact reduction capabilities observed with the new iMAR algorithm in our study may translate into comparable benefits for head and neck examinations, where dental materials remain a major source of clinically significant image degradation.

The following study limitations must be acknowledged. First, there are inherent limitations of our experimental ex vivo animal study, limiting the transfer of results to humans. Also, our bovine femur had no adjacent soft tissue, which may further impact the generalizability to human in vivo scanning. Second, we implanted only 3 trochanteric fixation nails made from titanium-based alloys, which are relatively thin, hereby not fully exploiting the effect of the new algorithm for other and larger devices. Third, the new iMAR algorithm was not compared with other metal artifact reduction algorithms from other vendors, but is limited to photon-counting detector CT from one vendor only. Fourth, we evaluated only a single, vendor-recommended iMAR preset. Prior studies have shown that vendor-recommended settings are not necessarily optimal for all implant types and artifact severities; therefore, further studies are warranted to evaluate the performance of different iMAR presets and parameter settings. Fifth, the low number of femurs included and the number of measurements limit statistical power. Also, despite predefined criteria and standardized ROI placement, a certain degree of reader-dependent subjectivity in ROI selection cannot be completely excluded. Sixth, a separate subgroup analysis distinguishing hyperattenuating and hypoattenuating artifacts was not performed. Seventh, the potential of the new iMAR algorithm for other metal implants remains to be determined. In addition, the evaluated algorithm is currently a prototype version, not yet applicable in clinical routine. However, approval of the new algorithm for clinical use is anticipated in the near future. Finally, potential aseptic loosening of the metal implant was only simulated by drilling holes of different sizes.

In conclusion, we evaluated a new prototype iMAR algorithm designed for photon-counting detector CT, which includes spectral image information to generate generic enhanced prior information. Our experimental results using bovine femurs with titanium implants demonstrate that the new iMAR algorithm reduces metal-related artifacts to a larger extent also in the close vicinity of the implants, while not introducing new artifacts. This, this algorithm has the potential to enable an enhanced appreciation of the bone-metal-interface area for diagnosing critical disease such as aseptic loosening with greater confidence and accuracy, which needs confirmation in future patient studies.
